# Wide-Antimicrobial Spectrum of Picolinium Salts

**DOI:** 10.3390/molecules25092254

**Published:** 2020-05-11

**Authors:** Sarka Salajkova, Marketa Benkova, Jan Marek, Radek Sleha, Lukas Prchal, David Malinak, Rafael Dolezal, Kristina Sepčić, Nina Gunde-Cimerman, Kamil Kuca, Ondrej Soukup

**Affiliations:** 1 Biomedical Research Center, University Hospital Hradec Kralove, Sokolska 581, 500 05 Hradec Kralove, Czech Republic; sarka.salajkova@img.cas.cz (Sa.S.); Marketa.Benkova@fnhk.cz (M.B.); jan.marek@fnhk.cz (J.M.); lukas.prchal@fnhk.cz (L.P.); david.malinak@uhk.cz (D.M.); rafael.dolezal@uhk.cz (R.D.); 2 Department of Genome Integrity, Institute of Molecular Genetics of the Czech Academy of Sciences, Videnska 1083, 142 20 Prague, Czech Republic; 3 Department of Epidemiology, University of Defence in Brno, Trebesska 1575, 500 05 Hradec Kralove, Czech Republic; radek.sleha@unob.cz; 4 Department of Chemistry, Faculty of Science, University of Hradec Kralove, Rokitanskeho 62, 500 03 Hradec Kralove, Czech Republic; 5 Department of Biology, Biotechnical Faculty, University of Ljubljana, Jamnikarjeva 101, 1000 Ljubljana, Slovenia; Kristina.Sepcic@bf.uni-lj.si (K.S.); Nina.Gunde-Cimerman@bf.uni-lj.si (N.G.-C.)

**Keywords:** picolinium salts, quaternary ammonium compounds, surfactant, critical micellar concentration, antimicrobial activity, cytotoxicity

## Abstract

Nosocomial infections, which greatly increase morbidity among hospitalized patients, together with growing antibiotic resistance still encourage many researchers to search for novel antimicrobial compounds. Picolinium salts with different lengths of alkyl chains (C_12_, C_14_, C_16_) were prepared by Menshutkin-like reaction and evaluated with respect to their biological activity, i.e., lipophilicity and critical micellar concentration. Picolinium salts with C_14_ and C_16_ side chains achieved similar or even better results when in terms of antimicrobial efficacy than benzalkoniums; notably, their fungicidal efficiency was substantially more potent. The position of the methyl substituent on the aromatic ring does not seem to affect antimicrobial activity, in contrast to the effect of length of the *N*-alkyl chain. Concurrently, picolinium salts exhibited satisfactory low cytotoxicity against mammalian cells, i.e., lower than that of benzalkonium compounds, which are considered as safe.

## 1. Introduction

Surfactants based on quaternary ammonium salts (QASs) are widely used from academic establishments to industry [1,2,3,4]. Because of their surface-active properties [5,6,7], they are commonly used as emulsifiers, moisturizers [8] and cleaning agents, as well as ointment disinfectants, pre-operative hand cleaning agents and topical hand sanitizers [9]. QASs can also be utilized as potential hydrolytic micellar catalysts to accelerate chemical decomposition, and such compounds can find uses as decontaminants [10,11,12,13] or in the preparation and coating of nanoparticles [14].

Most importantly, QASs have been used as disinfectants for years. The correct practice of disinfectants or their cocktail use within hospitals has long been considered the most appropriate first-line defense for decreasing the incidence of nosocomial infections and minimizing the prescription of antibiotics [1,15,16,17,18,19]. The ordinarily applied QASs are derivatives of benzalkonium, benzoxonium, pyridinium or cetrimonium salts [11].

**Figure 1:**
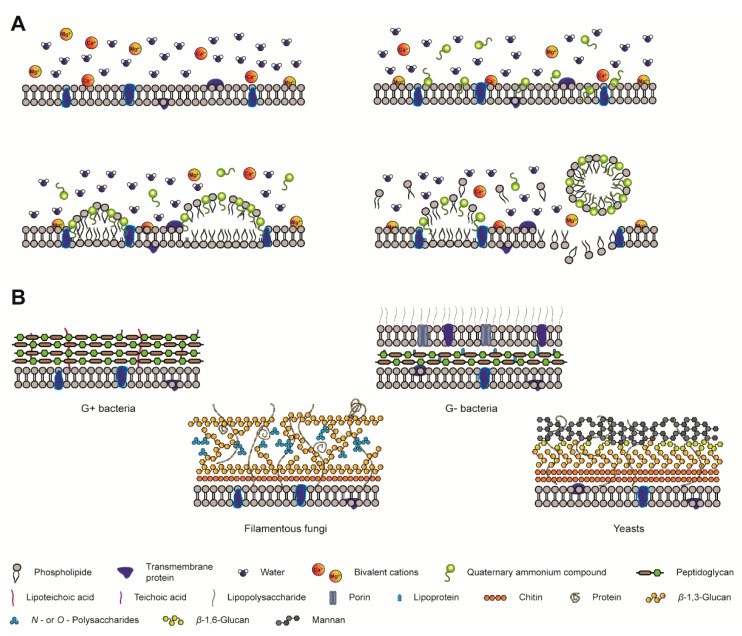
Cidal effect of QAS. Disintegration mechanism of the membrane by QAS attachment on to the membrane, followed by incorporation of side chains into the membrane resulting in membrane rupture and cytosol leakage (**A**). Fundamental composition of cell walls (**B**).

One of the most beneficial features of QASs is their cytotoxic effect at very low concentration to a broad spectrum of microorganisms, including bacteria, fungi, parasites and enveloped viruses, while showing relatively low toxicity to eukaryotic cells [20,21]. QASs generally act by disruption of the cytosolic membrane and outer lipid bilayer via replacement of bivalent ions and attachment to the membrane, followed by the incorporation of the hydrophobic side chain into the phospholipid bilayer and the creation of a mixed micelle with the phospholipids, resulting ultimately in cytosol leakage (Figure 1A) [10,18,22]. Whereas bacterial membranes are composed predominantly of negatively charged phospholipids, eukaryotic membranes mostly contain zwitterionic lipids and cholesterol [23,24,25]. Such a difference in composition is a good precondition for a selective effect against microbes, and therefore, QAS should exhibit selectivity to microorganisms over eukaryotic cells [4,15,25]. Indeed, selectivity is obvious even among microorganisms. Gram-positive (G+) bacteria are more sensitive than Gram-negative (G-). The increased resistance of G- is most likely a consequence of the presence of the outer membrane layer which grants the cells stronger physicochemical protection (Figure 1B) [18,25]. Interestingly, the relationship between the alkyl side-chain length and antibacterial activity is nonlinear. Optimal bioactivity is exhibited by C_12_–C_14_ chains for G+, and C_14_–C_16_ chains for G- bacteria [5,15,18,26]. However, the lipophilicity of the side chains seems to be a crucial factor [4,27], although the precise mechanism of action and structure–activity relationship is not yet fully understood.

Picolinium salts are pyridinium derivatives and can be prepared by modification of the Menshutkin reaction commonly used for the preparation of QAS [18,26,28,29,30,31]. Picolinium salts have, in many cases, been used as intermediates for the preparation of donor-acceptor molecules for the construction of promising photo- and electro-active materials [29,32,33,34,35,36,37,38]. In addition, their typical physicochemical features such as surface activity and micellization have been intensively studied in the line of their possible application [39,40,41,42,43,44]. For instance, *N*-(*n*-hexadecyl)- and *N*-(*n*-heptadecyl)-3-methylpyridinium bromide or 1-methyl-3-octylpyridinium alkyl sulfate were found to be effective for micelle-based drug delivery to improve drug solubility and the bioavailability of anionic drugs and metal complexes [28,43]. The Crooks’ group systematically investigated mono- and bis-picolinium derivatives as promising antagonists of neuronal nicotinic acetylcholine receptors, mediating nicotine-evoked dopamine release [45,46,47,48,49]. Although the antimicrobial activities of quaternary ammonium salts are generally known, the published results regarding the antimicrobial activities of picolinium salts are inconsistent. Several publications have reported an effect of some picolinium representatives, such as 1-dodecyl-4-methylpyridinium bromide [42], 1-dodecyl-3-methylpyridinium bromide [30], 3-methyl-1-tetradecylpyridinium bromide and 1-hexadecyl-3-methylpyridinium bromide [43], against *Micrococcus luteus* [30], *Staphylococcus aureus* [30,31,42,43], *Enterobacter aerogenes* [30], *Escherichia coli* [30,31,42,43], *Klebsiella pneumonia* [43] and *Pseudomonas æruginosa* [42]. However, there has been no study systematically evaluating the antimicrobial effectiveness of ortho, meta and para *N*-alkylpicolinium salts differing in their alkyl chain length. Furthermore, wide-spectrum efficacy against yeasts, fungi and viruses is unknown.

Therefore, the aim of this study is to systematically describe the biological properties of a picolinium series on a wide set of microorganisms, including bacteria, fungi and viruses. Besides evaluating the basic physicochemical parameters, we focused on the antimicrobial action in an attempt to elucidate the structure-activity relationship against various microbes and against mammalian cells for selectivity reasons.

## 2. Results and Discussion

### 2.1. Synthesis and Physicochemical Properties of Picolinium Salts

**Scheme 1:**
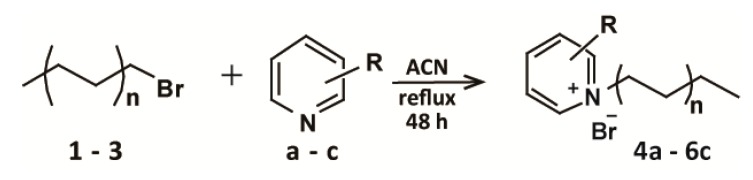
Preparation of alkyl chain compounds; *n* = 5, 6, 7; R = CH_3_.

Surfactants **4a**–**6c** were prepared in one step by Menshutkin-like reaction. The reaction conditions were chosen to facilitate the bimolecular nucleophilic substitution of the corresponding bromoalkane **1**–**3** with the appropriate methylpyridine isomers **a**–**c** in acetonitrile (ACN) (Scheme 1) [18]. The crude products were crystallized from diethyl ether to afford **4**–**6** in moderate to high yields (Table 1).

**Figure 2:**
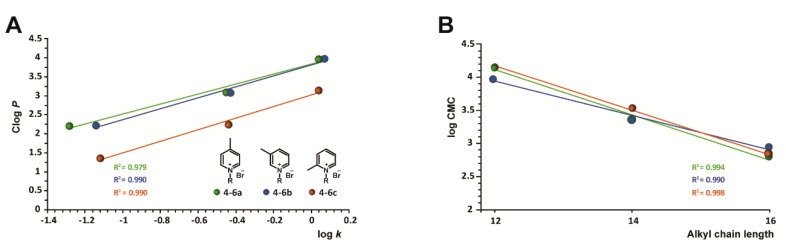
Correlation between compound structure and physicochemical properties. Graphs show the lipophilicity of the prepared compounds and their dependency on chain elongation (**A**). Stauff-Klevens plots of log CMC and extended chain of surfactants (**B**).

Lipophilicity is an important molecular descriptor governing the intercalation of the QAS side chain into the cell membrane, with its subsequent disruption and cell death. The lipophilicity of the compounds was estimated by chromatographic study using a simple HPLC-MS method with isocratic elution, as described previously [18,26]. The expected linear relationship between the prolongation of the alkyl chain of QAS **4a**–**6c** and the increasing lipophilicity driven by the length of the alkyl chain was observed with a high correlation coefficient (R^2^ < 0.97; values are listed in Figure 2). The position of the methyl group on the aromatic ring slightly affects the lipophilicity of the molecules. The position of the methyl group in the para- or meta- position did not result in any change on cLog *p*; however, a noticeable effect was observed by introucing this group into the ortho- position. Thus, the lipophilicity of the picolinium compounds increased in the order *o*-picolinium (**6a**–**c**) < *m*-picolinium (**5a**–**c**) = *p*-picolinium (**4a**–**c**).

A specific characteristic of surfactants is their ability to create micelles characterized as the critical micelle concentration (CMC) [4,11,50,51]. CMC was measured using a conductometric method, and the parameters of the experiment were set according to our previous investigations [9,26]. Elongation of the alkyl chains decreases the CMC value according to the Traube rule (Figure 2). Furthermore, the position of the methyl group in the hydrophilic head of the compounds has a negligible influence on micelle formation, which is clear proof that the tail length is the dominant factor for micelle creation. Pernak et al. demonstrated a relationship between the CMC and the antibacterial properties of 1-alkyl-3-alkylthiomethylimidazolium chlorides [52,53]. The published results imply that the micelle formation enhances the local concentration of surfactant with its cationic charge, leading to a stronger interaction with the negatively charged net of the cell membrane [54,55].

However, it is still not clear how micelle formation affects the antimicrobial properties, and the published results are inconsistent in this context. The biological activity seems to be not affected directly by the structure of substituents attached to the polar heads, but rather, by the morphology of aggregates, such as the size, which is enhanced with increasing the alkyl chain length [56]. In line with this knowledge, compounds **4a**–**6c** were used in the subsequent biological evaluation far below their CMC.

### 2.2. Antimicrobial Activity of Picolinium Salts

**Figure 3:**
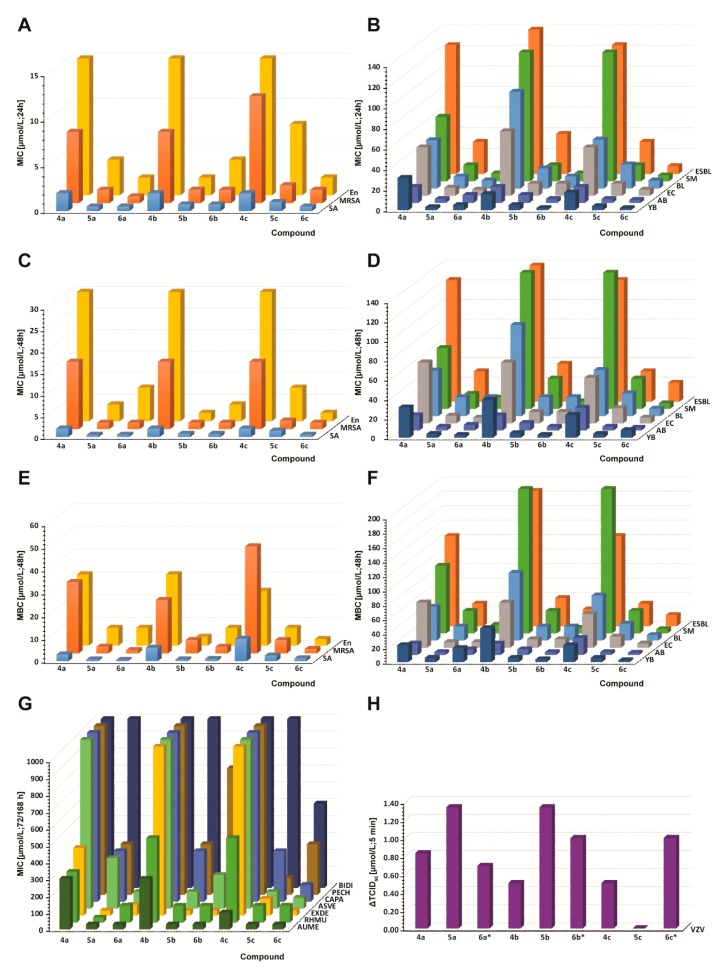
The biological assessment of prepared picolinium salts **4a–6c**. Minimum inhibitory concentration (MIC) after 24 h or 48 h incubation against Gram-positive (**A** or **C**) and Gram-negative bacteria (**B** or **D**). Minimum bactericidal concentration (MBC) after 48 h treatment against Gram-positive (**E**) and Gram-negative bacteria (**F**). Minimum inhibitory concentrations (MIC) determined on a panel of four yeasts and three filamentous fungi after 72 h (yeasts)/168 h (filamentous fungi) incubation (**G**). Virucidal efficacy against varicella zoster virus (VZV); 5 min exposure to 0.01%; or 0.005%; expressed as ΔTCID_50_ (**H**). Complete data are listed in Tables S1, S2 and S3.

The prepared picolinium salts showed strong antimicrobial properties below their CMC (Figure 3), as well as a broad spectrum of activities (Figure S2) against chosen microorganisms, including bacteria, fungi and viruses, while having simultaneously satisfactory cytotoxicity for mammalian cells. The results were compared with the activity of benzalkonium salts, which are considered the gold standard of quarternary ammonium salt-based disinfectants (data are shown in Supplementary Information, SI).

The antibacterial properties were evaluated as the minimum inhibitory and bactericidal concentrations (MIC and MBC, respectively) for nine bacterial strains, namely the three G+ strains, *Staphylococcus aureus* C1947 (SA), methicillin-resistant *Staphylococcus aureus* C1926 (MRSA), and *vancomycin-resistant enterococci* S2484 (En), and the six G- strains, *Acinetobacter baumannii* J3474 (AB), *Escherichia coli* A1235 (EC), *Klebsiella pneumoniae* C1950 (BL), extended-spectrum β-lactamase-producing *Klebsiella pneumoniae* C1934 (ESBL), *Stenotrophomonas maltophilia* J3552 (SM), and *Yersinia bercovieri* CNCTC6230 (YB). The quaternary ammonium salts with C_12_ or C_14_ alkyl chain lengths are considered the most efficacious against G+ and G− bacteria, respectively. Interestingly, the antibacterial effect against each strain, as well as the width of the antibacterial spectrum, was enhanced with increasing the length of the side chains C_12_ < C_14_ < C_16_. Picolinium salts have a similar trend of activity towards both G−and G+ bacteria (Figure 3A–D), yet the level of efficacy on those bacteria types is considerably different. The MIC and MBC for individual bacterial strains are listed in Table S1 (in SI).

Picolinium salts with a C_12_ side-chain showed lower activity, particularly against enterococci. Both C_14_ and C_16_ derivatives (**5a**–**6b**) exhibited very good efficacy against the tested G+ strains, with comparable or better antibacterial activity than that of benzalkonium salts (**5B, 6B**) (Figure S2). Stronger activities than those of benzalkonium salts were shown against *Staphylococcus aureus* by compounds with a C_14_ or C_16_ side chain. In the case of MRSA, the activities of compounds **5a**–**6b** were similar to those of benzalkonium salts. The strains of G+ bacteria were sensitive to the test compounds in the order: SA > MRSA > En.

The antibacterial activities against G− bacteria were significantly lower. The resistance of G− bacteria to antimicrobial agents presumably arises from the higher lipid content of the cell membrane and the presence of the outer phospholipid membrane layer with proteoglycans, which serves as a stronger physicochemical barrier [18,25,54,55,57]. The most promising activity was exhibited by compounds with a C_16_ alkyl chain, followed by those with C_14_, which attained similar activity as the standards (**5B**, **6B**). These analogues also have broad-spectrum efficacy, like benzalkonium salts (see SI). C_12_-derivatives had lower antibacterial effects, and poor activity in the case of *Stenotrophomonas maltophilia* and both Klebsiella strains. Overall, varying the position of the methyl group on the aromatic ring had a negligible effect on antibacterial efficacy compared to the elongation of the alkyl chain. Our results correspond to published data by Viscari et al.; those authors compared the antibacterial efficiency of 1-dodecyl-4-methylpyridinium bromide (**4a**) and *N*-benzyl-*N*,*N*-dimethyl-*N*-dodecylammonium bromide (**4B**) as standards against *Staphylococcus aureus*, *Escherichia coli* and *Pseudomonas æruginosa* [42].

The antifungal efficiencies were assessed as the minimum inhibitory concentrations (MIC) for seven fungi: four yeast strains, i.e., *Candida parapsilosis sensu stricto* EXF-8411 (CAPA), *Rhodotorula mucilaginosa* EXF-8417 (RHMU), *Exophiala dermatitidis* EXF-8470 (EXDE), *Aureobasidium melanogenum* EXF-8432 (AUME); and the three filamentous fungi, i.e., *Bisifusarium dimerum* EXF-8427 (BIDI), *Penicillium chrysogenum* EXF-1818 (PECH) and *Aspergillus versicolor* EXF-8692 (ASVE).

A similar relationship was observed between antifungal activity and picolinium side-chain prolongation as for antibacterial activity, i.e., the increasing trend of efficiency was with elongation of the side-chain C_12_ < C_14_ < C_16_. All prepared QASs had moderate to very good efficacy against *Aureobasidium melanogenum, Rhodotorula mucilaginosa* and *Exophiala dermatitidis*, while the remaining tested fungi showed only minor sensitivity to compounds bearing a C_12_ chain (**4a**–**c**). Picolinium salts **5a**–**6c** were efficient against a whole spectrum of selected fungi, even more so than benzalkonium salts. Again, the position of the methyl substituent on the aromatic ring does not seem to affect antifungal activity significantly compared to the effect of the alkyl chain length. MIC for individual fungal strains are listed in Table S2 (in SI).

Finally, the antiviral activity against varicella zoster virus (VZV) was determined in order to evaluate the width of the antimicrobial spectrum. The reduction factors, expressed as the change of values of 50% tissue culture infectious doses (logTCID_50_) of viral titer before and after 5 min exposure time to compounds being tested are summarized in Table S3 (see SI). The efficacy of the antiviral activity of antiviral drugs should exhibit at least a reduction factor of 4 in the virus titer. However, none of our compounds or standards achieved such a result. The highest effect against VZV was manifested by benzalkonium salts with C_12_ alkyl chain (reduction factor = 4.67), and from the picolinium series **5a** and **5b**, which exerted some activity at a concentration of 0.01% (reduction factor = 1.34). Predictably, higher antiviral activity could be expected at higher concentrations, i.e., typically those used in practice in the case of QASs, against enveloped viruses. Unfortunately, no SAR can be deduced, since C_16_ analogues, due to their limited solubility, were tested at half the concentration (0.005%) of C_12_ and C_14_ analogues (0.01%).

### 2.3. Cytotoxic Effect on Mammalian Cells

**Figure 4:**
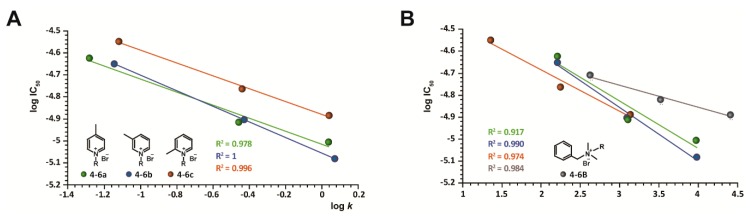
Cytotoxicity of prepared QASs **4a**–**6c** (**A**) and their comparison with benzalkonium salts **4**–**6B** (**B**).

A cell viability assay (the MTT assay) of the prepared compounds was performed on eukaryotic mammalian hamster ovarian cells (CHO-K1), and is expressed as IC_50_. In contrast to the cidal effects of the compounds, their cytotoxic potential on eukaryotic cells was linearly dependent on their lipophilicity. Figure 4 shows a significant trend that side chain elongation relates to increasing lipophilicity of the compound and higher cytotoxic effect, facilitated by better penetration into the cell membrane. The influence of the substituent position on the aromatic ring was more pronounced in comparison to the cidal effects, with cytotoxicity increasing in the order *o*-picolinium < *p*-picolinium < *m*-picolinium. The cytotoxicity, expressed as the IC_50_ for individual compounds, is listed in Table S4 (in SI).

The comparison of picolinium and benzalkonium salts with 3-hydroxypiridinium [18] clearly proved that the introduction of lipophobic substituents on pyridine contributes to the overall lipophilicity of compounds and increases their cytotoxic potential (Figure 4B). Moreover, the introduction of the hydroxyl substituent resulted in higher hydrophilicity of the whole structure and reduced cytotoxicity.

In summary, the cytotoxicity is clearly based on the lipophilicity of the compound in the way that the increasing lipophilicity causes higher toxic potential. However, the alkyl chain showed the major impact of the overall lipophilicity of the compounds; the minor contribution of the methyl substituent on aromatic ring was observed in the case of compounds **4a**–**6c**. Nevertheless, picolinium salts show a similar effect on cell viability as the benzalkoniums, which are considered to be nontoxic surfactants.

## 3. Materials and Methods 

### 3.1. General Synthetic Data

All commercial reagents for synthesis were purchased as reagent grade from Sigma-Aldrich (Prague, Czech Republic) and used without further purification. Anhydrous solvents for synthesis were purchased from Sigma-Aldrich (Czech Republic), and the others were purchased from Penta chemicals Co or VWR International (Prague, Czech Republic). Acetonitrile, methanol and formic acid for LC-MS analyses were obtained from Sigma-Aldrich in LC-MS grade purity (Czech Republic). Ultrapure water was produced by Barnstead Smart2Pure 3 UV/UF apparatus (ThermoFisher Scientific, Bremen, Germany). Thin-layer chromatography was carried out on Merck silica gel 60 F_254_ analytical plates (Prague, Czech Republic); detection was accomplished with phosphomolybdic acid stain (10 g PMA in 100 mL EtOH), or ultraviolet light (254 nm lamp). Uncorrected melting points of prepared compounds were determined by Melting Point Apparatus—Stuart SMP30 (Melting Point Apparatus – Stuart SMP30, Eaton, United Kingdom).

^1^H NMR and ^13^C NMR spectra of the prepared compounds were recorded at ambient temperature on a Varian S500 spectrometer (499.87 MHz for ^1^H and 125.71 MHz for ^13^C, Varian, Palo Alto, CA, USA). The NMR spectra were processed with MestReNova 12.0.4-22023, 2018 Mestrelab Research S.L. The chemical shift values for ^1^H and ^13^C NMR spectra are reported in ppm (δ) relative to residual solvent peak DMSO (δ_H_ = 2.55 ppm, δ_C_ = 39.52 ppm). For ^1^H, δ are given in parts per million (ppm) relative to solvent, and the coupling constants (*J*) are expressed in Hertz (Hz). Signals are quoted as s = singlet, d = doublet, t = triplet or m = multiplet.

The HPLC-MS analysis of intermediates and final products was performed using a Dionex UltiMate 3000 analytical system coupled with a Q Exactive Plus hybrid quadrupole-orbitrap spectrometer (ThermoFisher Scientific, Bremen, Germany) according to a gradient method using a Waters Atlantis dC18 (2.1 × 100 mm/3 μm; Waters, Ireland) column previously described by Benkova et al. (2019) [26]. The purity of samples was determined from UV chromatograms recorded at a wavelength of 254 nm, and the HRMS results were determined from the total ion current spectra.

#### 3.1.1. General Procedure for the Synthesis of Quaternary Ammonium Salts.

Appropriate bromide analogues (0.25 mmol, 1 eq) were dissolved in ACN (0.6 mL), and the corresponding amine (1.2 eq) was added. The reaction mixture was stirred under reflux for 48 h, and the solvent was evaporated under reduced pressure. The crude products were purified by crystallization from diethyl ether, filtered, washed with diethyl ether at ambient temperature, and allowed to dry to obtain corresponding products in yields 50–98%.

*1-Dodecyl-4-methylpyridinium bromide* (**4a**). Compound **4a** was obtained as white crystals (50%); m.p. 41.4 °C–42.8 °C. ^1^H-NMR (500 MHz, DMSO) δ (ppm): 8.99 (d, *J* = 6.6 Hz, 2H), 8.00 (d, *J* = 6.5 Hz, 2H), 4.55 (t, *J* = 7.4 Hz, 2H), 2.60 (s, 3 H), 1.92–1.83 (m, 2H), 1.30–1.17 (m, 18 H), 0.84 (t, *J* = 6.9 Hz, 3H). ^13^C-NMR (126 MHz, DMSO) δ (ppm): 158.69, 143.69, 128.30, 59.80, 31.26, 30.57, 28.97, 28.88, 28.76, 28.67, 28.35, 25.34, 22.06, 21.33, 13.92. HRMS (HESI^+^): calculated for [M^+^] C_18_H_32_N^+^ (*m*/*z*) 262.2529; found 262.2527.

*4-Methyl-1-tetradecylpyridinium bromide* (**5a**). Compound **5a** was obtained as white crystals (95%); m.p. 60.4 °C–62.3 °C. ^1^H-NMR (500 MHz, DMSO) δ (ppm): 8.96 (d, *J* = 6.7 Hz, 2H), 7.99 (d, *J* = 6.6 Hz, 2H), 4.53 (t, *J* = 7.4 Hz, 2H), 2.60 (s, 3H), 1.91–1.80 (m, 2H), 1.36–1.09 (m, 22H), 0.84 (t, *J* = 6.9 Hz, 3H). ^13^C-NMR (126 MHz, DMSO) δ (ppm): 158.71, 143.68, 128.31, 59.84, 31.27, 30.56, 29.03, 29.01, 28.99, 28.97, 28.89, 28.77, 28.68, 28.35, 25.35, 22.07, 21.34, 13.93. HRMS (HESI^+^): calculated for [M^+^] C_20_H_36_N^+^ (*m*/*z*) 290.2842; found: 290.2837.

*1-Hexadecyl-4-methylpyridinium bromide* (**6a**). Compound **6a** was obtained as white crystals (98%); m.p. 79.9 °C–81.9 °C. ^1^H-NMR (500 MHz, DMSO) δ (ppm): 8.98 (d, *J* = 6.7 Hz, 2H), 8.00 (d, *J* = 6.6 Hz, 2H), 4.54 (t, *J* = 7.4 Hz, 2H), 2.61 (s, 3H), 1.95–1.78 (m, 2H), 1.44–1.13 (m, 26H), 0.84 (t, *J* = 6.9 Hz, 3H). ^13^C-NMR (126 MHz, DMSO) δ (ppm): 158.69, 143.68, 128.30, 59.81, 31.26, 30.56, 29.02, 28.97, 28.89, 28.77, 28.67, 28.36, 25.34, 22.06, 21.32, 13.91. HRMS (HESI^+^): calculated for [M^+^] C_22_H_40_N^+^ (*m*/*z*): 318.3155; found: 318.3149.

*1-Dodecyl-3-methylpyridinium bromide* (**4b**). Compound **4b** was obtained as white crystals (80%); m.p. 34.0 °C–36.0 °C. ^1^H-NMR (500 MHz, DMSO) δ (ppm): 9.09 (s, 1H), 8.98 (d, *J* = 6.0 Hz, 1H), 8.46 (d, *J* = 8.0 Hz, 1H), 8.10–8.04 (m, 1H), 4.60–4.54 (m, 2H), 2.50 (s, 3H), 1.96–1.87 (m, 2H), 1.48–1.14 (m, 18H), 0.84 (t, *J* = 6.9 Hz, 3H); ^13^C-NMR (126 MHz, DMSO) δ (ppm): 145.69, 144.22, 141.94, 138.68, 127.29, 60.49, 31.26, 30.62, 28.97, 28.89, 28.75, 28.67, 28.36, 25.37, 22.06, 17.83, 13.92. HRMS (HESI^+^): calculated for [M]^+^ C_18_H_32_N^+^ (*m*/*z*): 262.2529; found: 262.2525.

*3-Methyl-1-tetradecylpyridinium bromide* (**5b**). Compound **5b** was obtained as white crystals (95%); m.p. 72.3 °C–74.3 °C. ^1^H-NMR (500 MHz, DMSO) δ (ppm): 9.08 (s. 1H), 8.97 (d, *J* = 6.0 Hz, 1H), 8.46 (d, *J* = 8.0 Hz, 1H), 8.09–8.04 (m, 1H), 4.59–4.54 (m, 2H), 2.50 (s, 3H), 1.95–1.87 (m, 2H), 1.55–0.97 (m, 22H), 0.85 (t, *J* = 6.9 Hz, 3H); ^13^C-NMR (126 MHz, DMSO) δ (ppm): 145.70, 144.21, 141.94, 138.70, 127.29, 60.52, 31.27, 30.62, 29.03, 29.01, 28.99, 28.98, 28.90, 28.75, 28.68, 28.37, 25.38, 22.07, 17.84, 13.92. HRMS (HESI^+^): calculated for [M]^+^ (*m*/*z*) C_20_H_36_N^+^ 290.2842; found: 290.2835.

*1-Hexadecyl-3-methylpyridinium bromide* (**6b**)**.** Compound **6b** was obtained as white crystals (96%); m.p. 70.8 °C–72.6 °C. ^1^H-NMR (500 MHz, DMSO) δ (ppm): 9.09 (s, 1H), 8.98 (d, *J* = 6.0 Hz, 1H), 8.46 (d, *J* = 8.0 Hz, 1H), 8.10–8.03 (m, 1H), 4.60–4.53 (m, 2H), 2.50 (s, 3H), 1.96–1.86 (m, 2H), 1.48–1.14 (m, 26H), 0.84 (t, *J* = 6.9 Hz, 3H). ^13^C-NMR (126 MHz, DMSO) δ (ppm): 145.69, 144.21, 141.94, 138.68, 127.28, 60.50, 31.26, 30.62, 29.02, 28.97, 28.90, 28.75, 28.67, 28.37, 25.38, 22.06, 17.82, 13.91. HRMS (HESI^+^): calculated for [M^+^] C_22_H_40_N^+^ (*m*/*z*): 318.31553; found: 318.3149.

*1-Dodecyl-2-methylpyridinium bromide* (**4c**). Compound **4c** was obtained as white crystals (88%); m.p. 121.9 °C–123.9 °C. ^1^H-NMR (500 MHz, DMSO) δ (ppm): 9.07 (d, *J* = 6.7 Hz, 1H), 8.51–8.45 (m, 1H), 8.07 (d, *J* = 7.8 Hz, 1H), 8.01–7.95 (m, 1H), 4.59–4.54 (m, 2H), 2.85 (s, 3H), 1.88 – 1.79 (m, 2H), 1.42–1.13 (m, 18H), 0.84 (t, *J* = 6.9 Hz, 3H); ^13^C-NMR (126 MHz, DMSO) δ (ppm): 155.04, 145.33, 145.00, 129.92, 125.52, 57.19, 31.25, 29.36, 28.97, 28.96, 28.90, 28.80, 28.67, 28.43, 25.63, 22.05, 19.55, 13.91. HRMS (HESI^+^): calculated for [M]^+^ C_18_H_32_N^+^ (*m*/*z*): 262.25293; found: 262.2525.

*2-Methyl-1-tetradecylpyridinium bromide* (**5c**). Compound **5c** was obtained as white crystals (84%); m.p. 125.7 °C–127.4 °C. ^1^H-NMR (500 MHz, DMSO) δ (ppm): 9.06 (d, *J* = 6.0 Hz, 1H), 8.51–8.45 (m, 1H), 8.07 (d, *J* = 7.8 Hz, 1H), 8.01–7.95 (m, 1H), 4.59–4.52 (m, 2H), 2.85 (s, 3H), 1.88–1.77 (m, 2H), 1.44–1.04 (m, 22H), 0.84 (t, *J* = 6.9 Hz, 3H); ^13^C-NMR (126 MHz, DMSO) δ (ppm): 155.04 145.33, 145.00, 129.92, 125.52, 57.19, 31.25, 29.36, 29.02, 29.00, 28.97, 28.91, 28.81, 28.67, 28.43, 25.63, 22.05, 19.54, 13.91. HRMS (HESI^+^): calculated for [M]^+^ C_20_H_36_N^+^ (*m*/*z*): 290.28423, found: 290.2835.

*1-Hexadecyl-2-methylpyridinium bromide* (**6c**). Compound **6c** was obtained as white crystals (80%); m.p. 126.8 °C–128.8 °C. ^1^H-NMR (500 MHz, DMSO) δ (ppm): 9.02 (d, *J* = 6.1 Hz, 1H), 8.51–8.44 (m, 1H), 8.05 (d, *J* = 7.9 Hz, 1H), 8.00–7.94 (m, 1H), 4.57–4.50 (t, *J* = 6.8 Hz, 2H), 2.84 (s, 3H), 1.88–1.78 (m, 2H), 1.58–1.06 (m, 26 H), 0.85 (t, *J* = 6.8 Hz, 3H); ^13^C-NMR (126 MHz, DMSO) δ (ppm): 155.06, 145.32, 145.01, 129.93, 125.54, 57.23, 31.26, 29.36, 29.02, 28.98, 28.91, 28.81, 28.68, 28.44, 25.65, 22.07, 19.53, 13.93. HRMS (HESI^+^) calculated for [M]^+^ C_22_H_40_N^+^ (*m*/*z*): 318.31553; found: 318.3153.

#### 3.1.2. Determination of Lipophilicity Expressed as Clog k or Clog *p*

Capacity factors *k* of the final products were measured with the aforementioned HPLC-MS system using the isocratic method described by Benkova et al. (2019) [26], with the ratio of ACN:H_2_O (both with 0.1% formic acid *v*/*v*) changed to 73:27. The studied compounds were dissolved in methanol. Uracil (Sigma Aldrich, Steinheim, Germany) was used as a void volume marker (*t_0_*). Retention times were determined from mass spectrometry total ion current scans. Capacity factors *k* were calculated using the equation:

(1) $$k=\frac{t_{1}-t_{0}}{t_{0}}$$

where *t*_1_ = retention time of the analyte and *t*_0_ = void time. Uracil was used as a void volume marker. Lipophilicity expressed as Clog *p* was calculated using MarvinSketch (Marvin 17.21.0, ChemAxon, Budapest, Hungary, https://www.chemaxon.com), and the values were correlated to log *k* values. Lipophilicity was expressed as a dependence of log *k* to the calculated Clog *p*, and the results in the studied group were characterized by classical statistical criteria: coefficient of determination R^2^ (0.97+).

#### 3.1.3. Conductivity Measurements

Conductivity measurements of aqueous solutions of the prepared surfactants were carried out in triplicate on a Tristar Orion conductivity meter with conductivity cell 013005MD (Thermo Scientific, Waltham, MA, USA). The apparatus was controlled by the Navigator 21 software, using continuous data collection. The experiment was set up according to previous experience: the solutions were temperature-controlled at 45 ± 0.1 °C [9]. Aqueous stock solutions of surfactants were prepared at concentrations of 0.1500 mol/L for C_12_ homologs, 0.0200 mol/L for C_14_ homologs and 0.0150 mol/L for C_16_. For the determination of CMC, the aqueous stock solutions of surfactants were further diluted. The linear pump Lineomat (VEB MLW Labortechnik Ilmenau, Ilmenau, Germany) was used with a flow rate of 0.43 mL/min. An AREX stirrer (VELP Scientifica Srl, Milano, Italy) was used for continuous agitation, and conductivity was recorded every 3 s. The equation for the linear regression and the coefficient of determination was calculated for both linear parts, and the intersection of both axes was determined as the value of CMC (Figure S1).

### 3.2. The biological Evaluation of Picolinium Salts

#### 3.2.1. Evaluation of Antibacterial Activity

The antibacterial activities of the prepared compounds were investigated on nine bacterial strains, i.e., three Gram-positive (*Staphylococcus aureus* C1947, methicillin-resistant *S. aureus* C1926, vancomycin-resistant enterococci S2484), and six Gram-negative (*Escherichia coli* A1235, *Klebsiella pneumoniae* C1950, extended-spectrum β-lactamase-producing *K. pneumoniae* C1934, *Acinetobacter baumannii* J3474, *Stenotrophomonas maltophilia* J3552 and *Yersinia bercovieri* CNCTC 6230). The latter was ordered from the Czech National Collection of Type Cultures (CNCTC), while the others were mostly resistant clinical isolates of patients from the University Hospital in Hradec Kralove (Czech Republic).

The antibacterial properties and the minimum inhibitory (MIC) and minimum bactericidal (MBC) concentrations of the compounds tested were determined by the broth microdilution testing method modified according to standard M07-A11 [58] and the protocol published previously [26]. Mueller-Hinton broth (M-H broth, HiMedia, Mumbai, India) adjusted to pH 7.4 (±0.2) was used as the test medium. All compounds were dissolved in dimethyl sulfoxide (DMSO; Sigma Aldrich, St. Louis, MO, USA) so that the final concentration of DMSO did not exceed 1% in the test medium (also applied to growth control). The wells of the microtitration plates contained 200 μL of the M-H broth with two-fold serial dilutions of the compounds (500; 250; 125; 62.5; 31.25; 15.63; 7.81; 3.91; 1.95; 0.98; 0.49 μmol/L), and were inoculated with 10 μL of bacterial suspension. A bacterial inoculum in sterile water (B. Braun Medical s.r.o., Melsungen, Germany) was adjusted densitometrically to match 0.5 McFarland turbidity scale. The MIC values, defined as complete inhibition of bacterial growth, were read visually and verified by microscopy after 24 h and 48 h incubation at 37 °C ± 1 °C. The MBCs were established for all prepared compounds as the concentrations that provided ≥99.9% decrease in the number of colonies after subculturing a 10 μL aliquot from each well in 200 μL of fresh M-H broth. The total incubation time was 48 h. For comparison, standard reference compounds *N*-benzalkonium bromide (**4B**, **5B**, **6B**) were included in the corresponding concentrations.

#### 3.2.2. Evaluation of Antifungal Activity

The antifungal activity was evaluated on seven fungal strains, i.e., four yeasts (*Candida parapsilosis sensu stricto* EXF-8411, *Rhodotorula mucilaginosa* EXF-8417 and *Exophiala dermatitidis* EXF-8470, *Aureobasidium melanogenum* EXF-8432), and three filamentous fungi (*Bisifusarium dimerum* EXF-8427, *Penicillium chrysogenum* EXF-1818, and *Aspergillus versicolor* EXF-8692). The strains were obtained from the Ex Culture collection (in the frame of the Infrastructural Centre Mycosmo) at the Department of Biology, Biotechnical Faculty, University of Ljubljana, Ljubljana, Slovenia. All isolates were maintained on malt extract agar (MEA) prior to being tested.

The antifungal activities were evaluated using the agar-well diffusion test according to the adjusted protocol published by Zovko et al. [59]. The fungal strain in the exponential (log) phase of growth was inoculated into 10 mL of sterile liquid malt extract broth (MEB), and 100 µL of the prepared suspension was poured onto solid MEA and spread on the plate surface. After the suspension dried, the circles of agar (Φ = 5.5 mm) were cut out from the inoculated culture plates. Then, 50 µL of the prepared compounds diluted in DMSO was added into the wells in the agar plates. Each concentration (1, 0.5, 0.3, 0.1, 0.03, 0.01 mmol/L) of the compounds was tested three times using three separate agar plates. Plates were incubated at room temperature, and after 72 h (yeasts) or 7 days (filamentous fungi) of incubation, the minimum inhibitory concentrations (MICs), defined as the lowest concentrations which inhibit the visible growth of the microorganisms being tested, were determined. The diameters of the inhibition zones were measured with a ruler. The shortest distance (0.5 mm) from the outer margin of the well to the initial point of microbial growth was estimated as the inhibitory zone. The inhibitory activity of DMSO was also checked and found to be zero.

#### 3.2.3. Evaluation of Virucidal Activity

The virucidal activity of synthesized agents was evaluated using a strain of the varicella zoster virus (VZV) from the collection of VZV isolates at the Department of Epidemiology, Faculty of Military Health Sciences in Hradec Kralove, University of Defence in Brno, Czech Republic.

A quantitative suspension test was used to determine the virucidal activity of the tested compounds against VZV. Briefly, one part of the virus suspension was mixed with one part of fetal bovine serum diluted with phosphate-buffered saline (1:5.5) and eight parts of the prepared compounds or reference drugs **4B**, **5B**, **6B**. The mixtures were kept at room temperature for 5 min (exposure time). After the exposure period, the mixtures were serially diluted ten-fold with ice-cold DMEM. Then, 100 µL of each dilution were seeded into a 96-well microtiter plate with NHLF cells (ATCC, Manassas, VA, USA) and incubated at 37 °C in a 5% CO_2_ atmosphere until a cytopathic effect was detected, i.e., approximately 10–12 days. Six wells per sample dilution were inoculated. Virus-untreated controls with identical protein concentrations were also tested.

Virus titer was determined using the method of Spearman and Kaerber and expressed as log10 TCID_50_/mL including the standard [60]. According to the European Standard EN 14476 for virucidal activity of disinfection, a product should demonstrate at least a log10 reduction of 4 in virus titer, corresponding to 99.99% inactivation [61]. To differentiate between virus-induced cytopathogenic changes and the toxic effect caused by the tested compounds, the cell monolayer was monitored until the end of the virucidal activity testing period for morphological changes after exposure to the disinfectant solution.

#### 3.2.4. Cell Viability Assessment

Standard MTT assay (3-(4,5-dimethylthiazol-2-yl)-2,5-diphenyltetrazolium bromide, Sigma Aldrich, St. Louis, MO, USA) was used according to the manufacturer’s protocol on CHO-K1 cells (Chinese hamster ovary, ECACC, Salisbury, UK) in order to compare the cytotoxic effect of the studied compounds [26]. The cells were cultured according to ECACC recommended conditions and seeded at a density of 8000 per well. Briefly, the tested compounds were dissolved in DMSO and subsequently in the growth medium (F-12) supplemented with 10% FBS and 1% penicillin/streptomycin, so that the final concentration of DMSO did not exceed 0.5% (*v*/*v*). Cells were exposed to the tested compounds for 24 h. The medium was then replaced by a medium containing 0.5 mg/mL of MTT, and the cells were allowed to produce formazan for approximately another 3 h under surveillance. Thereafter, the medium with MTT was removed, and crystals of formazan were dissolved in DMSO (100 μL). Cell viability was assessed spectrophotometrically by the amount of formazan produced. Absorbance was measured at 570 nm with a 650 nm reference wavelength on Synergy HT (BioTek, Winooski, VT, USA). IC_50_ was then calculated from the control-subtracted triplicates using nonlinear regression (four parameters) in the GraphPad Prism 5.03 and 7.03 software (GraphPad Software Inc., San Diego, CA, USA). Final IC_50_ and SEM values were obtained as a mean of three independent measurements.

## 4. Conclusions

Picolinium salts with an aliphatic carbon chain (C_12_–C_16_) were prepared by one-step synthesis. The products were characterized by common analytical methods (NMR and HRMS) and their biological activities, lipophilicity and critical micellar concentration (CMC) were evaluated. The prepared picolinium salts showed good antimicrobial properties as well as broad-spectrum activity against a selected panel of microorganisms including bacteria, fungi and enveloped viruses below their CMC values. Their activities were compared with the antimicrobial effect of commonly used benzalkonium analogues with the same alkyl chain length. Picolinium salts with C_14_ and C_16_ side chains achieved similar or better results for antimicrobial efficacy than the reference benzalkoniums. Their antibacterial activity was comparable to that of selected standards on both G+ and G− bacteria, although against G− the antibacterial effect was weaker. The best activity was shown against *Staphylococcus aureus* (G+), while for G− strains, the greatest efficiency was observed against *Acinetobacter baumannii* and *Yersinia bercovieri.* Furthermore, they showed promising antifungal efficiency. Concurrently, the picolinium salts exhibited satisfactory low cytotoxicity against mammalian cells, i.e., comparable to that of benzalkonium compounds, which are considered as safe.

## Figures and Tables

**Table 1:** Prepared compounds with alkyl chains and their physicochemical properties.

Structure	Cpd ^a^	Chain Length	Yield [%]	m.p.^b^ [°C]	*k* ^c^	log *k*	Clog *p* ^d^	CMC ^e^ [μmol/L]
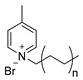 *n* = 5, 6, 7	**4a**	C_12_H_25_	50	121.9–123.9	0.05 ± 0.01	−1.28	2.21	14,010
	**5a**	C_14_H_29_	95	125.7–127.4	0.35 ± 0.01	−0.45	3.10	2330
	**6a**	C_16_H_33_	98	126.8–128.8	1.09 ± 0.01	0.04	3.98	600
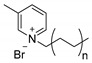 *n* = 5, 6, 7	**4b**	C_12_H_25_	80	34.0–36.0	0.07 ± 0.01	−1.14	2.21	9490
	**5b**	C_14_H_29_	95	72.3–74.3	0.37 ± 0.01	−0.43	3.10	2320
	**6b**	C_16_H_33_	96	70.8–72.6	1.18 ± 0.01	0.07	3.98	860
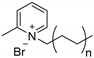 *n* = 5, 6, 7	**4c**	C_12_H_25_	88	41.4–42.8	0.08 ± 0.01	−1.12	1.36	14,160
	**5c**	C_14_H_29_	4	60.4–62.3	0.36 ± 0.02	−0.44	2.25	3460
	**6c**	C_16_H_33_	80	79.9–81.9	1.10 ± 0.01	0.04	3.14	650

^a^ Compound (Cpd), ^b^ Melting point (m.p.), ^c^ Capacity factor (*k)* was determined by an isocratic LC-MS method, ^d^ Marvin was used for drawing, displaying and characterizing chemical structures (Clog *p*), substructures and reactions, Marvin 17.21.0, ChemAxon (https://www.chemaxon.com), ^e^ Critical micellar concentration (CMP).

## Supplementary Materials

The following are available online at https://www.mdpi.com/1420-3049/25/9/2254/s1. Figure S1: Dependency of CMC-specific conductivity on concentration; Figure S2: The broad-spectrum antimicrobial activity; Table S1: Antibacterial activity; Table S2: Antifungal activity; Table S3: Antiviral activity; Table S4.: Cytotoxicity and calculated lipophilicity

## Abbreviations

AB	*Acinetobacter baumannii*
ACN	acetonitrile
ASVE	*Aspergillus versicolor*
AUME	*Aureobasidium melanogenum*
BIDI	*Bisifusarium dimerum*
BL	*Klebsiella pneumoniae*
CAPA	*Candida parapsilosis sensu stricto*
CMC	critical micelle concentration
EC	*Escherichia coli*
En	Vancomycin-resistant enterococci
ESBL	extended spectrum β-lactamase producing *Klebsiella pneumoniae*
EXDE	*Exophiala dermatitidis*
HPLC-MS	high-performance liquid chromatography coupled with mass spectrometry
HRMS	high resolution mass spectrometry
MIC	minimum inhibitory concentration
MBC	minimum bactericidal concentration;
MRSA	Methicillin-resistant *Staphylococcus aureus*
MTT	3-(4,5-dimethyl-2-thiazolyl)-2,5-diphenyl-2H-tetrazolium bromide
NMR	nuclear magnetic resonance
PECH	*Penicillium chrysogenum*
QAS	quaternary ammonium salts
RHMU	*Rhodotorula mucilaginosa*
SA	*Staphylococcus aureus*
SEM	standard deviation
SE	*Staphylococcus epidermis*
SM	*Stenotrophomonas maltophilia*
VZV	Varicella zoster virus
YB	*Yersinia bercovieri*
