# Supplementary material for: Wide-Antimicrobial Spectrum of Picolinium Salts
Source: Molecules. 2020 May 11;25(9):2254. doi: 10.3390/molecules25092254 (PMC7248777; doi:10.3390/molecules25092254)
Supplement: Supplementary file 1 [file molecules-25-02254-s001.pdf]

Table of content

**Figure S1.**	Dependency of CMC-specific conductivity on concentration	……	2
**Figure S2.**	The broad-spectrum antimicrobial activity	……	3
**Table S1.**	Antibacterial activity	……	5
**Table S2.**	Antifungal activity	……	6
**Table S3.**	Antiviral activity	……	7
**Table S4.**	Cytotoxicity and calculated lipophilicity	……	7
**Reference**		……	8

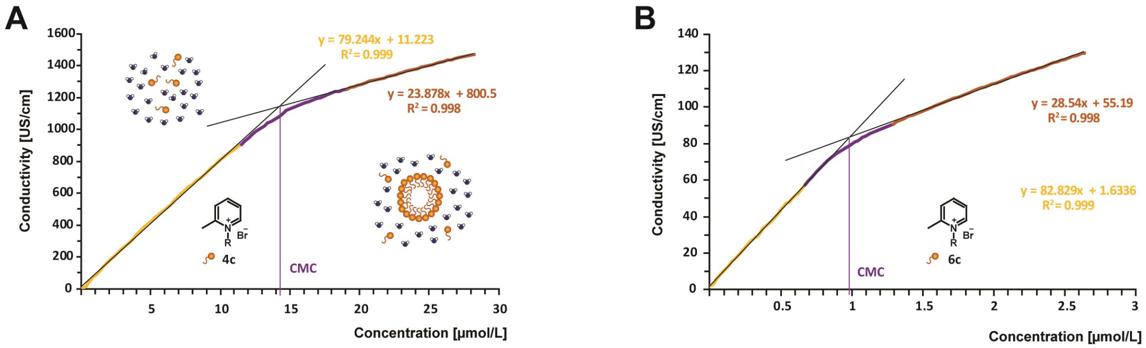

Figure S1. Principle of dependency of CMC-specific conductivity on o-picolinium salts actual concentration with respect to their alkych chain length (showed for C_{12} **A.**; for C_{16} **B**.). The change in the electrical conductance of aqueous ionic surfactant solutions at the CMC (inflection part, lila), which is a well-defined concentration of surfactant, where the degrees of surfactant ionizations below (part 1, yellow) and above (part 3, brown) of the CMC differ, on the assumption that the aqueous surfactant solutions obey Kohlrausch's law [1]. The linear path (part 1) exhibits the linear increase of conductivity on the concentration of cationic salt in the water, where the surfactant monomers behave as strong electrolytes. The reach of CMC represents the intersection point of part 1 and part 3. The exceeding of the CMC is followed by the linear path with a lower slope, where monomers and micelles exist in dynamic equilibrium (micelles are partially ionized).

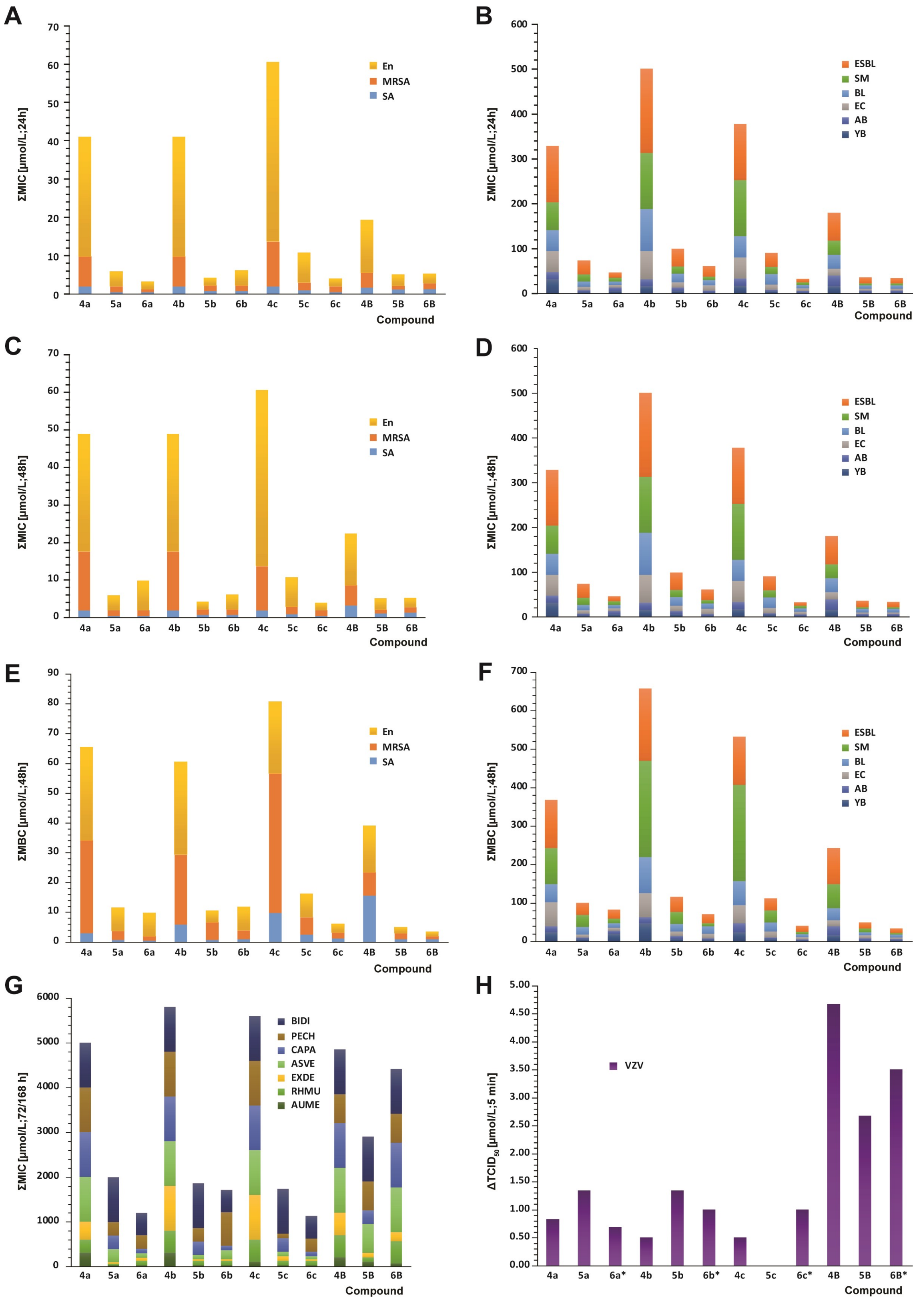

Figure S2. The broad-spectrum antimicrobial activity of prepared picolinium salts **4a** – **6c** and benzalkonium salts as standards **4** – **6B**. Minimum inhibitory concentrations (MIC) after

24h or 48h incubation against Gram-positive (**A.** or **C.**) and Gram-negative bacteria (**B.** or **D**). Minimum bactericidal concentration (MBC) after 48h treatment against Gram-positive (**E.**) and Gram-negative bacteria (**F.**). Minimum inhibitory concentrations (MIC) determined on a panel of four yeasts and three filamentous fungi after 72h (yeasts)/168h (filamentous fungi) incubation (**G.**). Virucidal efficacy against Varicella zoster virus (VZV); 5 min exposure to 0.01% or *0.005% compounds; expressed as $\Delta TCID_{50}$ (**H.**).

Table S1. Minimum inhibitory and bactericidal concentrations (MIC and MBC) of the prepared compounds **4a – 6c** and standard benzalkonium salts (**4B – 6B**). The activity was determined *in vitro* on a panel of three Gram-positive and Gram-negative strains. The prepared compounds, as well as benzalkonium salts, were also tested against multidrug-resistant *Pseudomonas aeruginosa*. However, none of the compounds showed any antibacterial properties against that bacterial strain.

	Microorganism	MIC [µmol/L]; 24h/48h incubation<br>MBC [µmol/L] ; 48h incubation											
		Compound											
		4a	5a	6a	4b	5b	6b	4c	5c	6c	4B[a]	5B[a]	6B[a]
Gram-positive	*Staphylococcus aureus* C1947 (SA)	1.95/<br>1.95	0.49/<br>0.49	0.49/<br>0.49	1.95/<br>1.95	0.74/<br>0.74	0.74/<br>0.74	1.95/<br>1.95	0.98/<br>1.47	0.49/<br>0.49	1.63/<br>3.26	1.14/<br>1.46	1.3/<br>1.3
		2.93	0.74	0.49	5.86	0.74	0.98	9.77	2.45	1.22	15.63	0.98	0.98
	Methicillin-rezistant *Staphylococcus aureus* C1926 (MRSA)	7.81/<br>15.63	1.47/<br>1.47	0.74/<br>1.47	7.81/<br>15.63	1.47/<br>1.47	1.47/<br>1.47	11.72/<br>15.63	1.95/<br>1.95	1.47/<br>1.47	3.91/<br>5.37	0.98/<br>0.98	1.47/<br>1.47
		31.25	2.93	1.47	23.44	5.86	2.93	46.88	5.86	1.95	7.81	1.95	0.98
	Vanckomycin-reristant *enterococci* S2484 (En)	31.25/<br>31.25	3.91/<br>3.91	1.95/<br>7.81	31.25/<br>31.25	1.95/<br>1.95	3.91/<br>3.91	46.88/<br>46.88	7.81/<br>7.81	1.95/<br>1.95	13.68/<br>13.68	2.93/<br>2.93	2.44/<br>2.44
		31.25	7.81	7.81	31.25	3.9	7.81	24.07	7.81	2.93	15.63	1.95	1.47
Gram-negative	*Yersinia bercovieri* CNCTC6230 (YB)	31.25/<br>31.25	2.93/<br>3.91	4.88/<br>3.93	15.63/<br>39.07	4.88/<br>4.88	1.95/<br>2.93	17.25/<br>23.44	3.905/<br>3.91	1.95/<br>7.81	15.63/<br>15.63	1.95/<br>1.95	1.95/<br>1.95
		23.44	5.86	19.53	46.875	5.86	3.91	23.44	5.86	2.45	15.63	1.95	1.95
	Acinetobacter baumannii J3474 (AB)	15.63/<br>15.63	3.91/<br>3.91	7.81/<br>5.86	15.63/<br>15.63	7.81/<br>7.81	3.91/<br>3.91	15.63/<br>23.44	3.91/<br>3.91	2.93/<br>2.93	23.44/<br>23.44	3.91/<br>3.91	3.91/<br>3.91
		15.63	3.91	7.81	15.63	7.81	3.91	23.44	3.91	2.93	23.44	5.86	3.91
	Escherichia coli A1235 (EC)	46.88/<br>62.5	7.81/<br>7.81	5.86/<br>5.86	62.5/<br>62.5	11.72/<br>11.72	11.72/<br>11.72	46.88/<br>46.88	11.72/<br>15.63	5.86/<br>5.86	15.63/<br>15.63	3.91/<br>3.91	3.91/<br>3.91
		62.5	7.81	7.81	62.5	11.72	11.72	46.875	15.63	5.86	15.63	7.81	3.91
	Klebsiella pneumoniae C1950 (BL)	46.88/<br>46.88	11.72/<br>19.53	7.81/<br>19.53	93.75/<br>93.75	19.53/<br>19.53	11.72/<br>19.53	47.5/<br>47.5	23.44/<br>23.44	7.81/<br>7.81	31.25/<br>31.25	7.81/<br>7.81	7.81/<br>7.81
		46.88	19.53	11.72	93.75	19.53	19.53	62.5	23.44	7.81	31.25	7.81	7.81
	Stenotrophomonas maltophilia J3552 (SM)	62.5/<br>62.5	15.63/<br>15.63	7.81/<br>11.72	125/<br>125	15.63/<br>15.63	7.81/<br>7.81	125/<br>250	15.63/<br>31.25	5.86/<br>5.86	31.25/<br>46.88	3.91/<br>3.91	3.91/<br>3.91
		93.75	35.25	11.7	250	31.25	7.81	250	31.25	5.86	62.5	9.77	3.91
	Extended-spectrum β-lactamase - producing *Klebsiella pneumonie* C1934 (ESBL)	125/<br>125	31.25/<br>31.25	11.72/<br>19.53	187.5/<br>187.5	39.07/<br>39.07	23.44/<br>23.44	125/<br>125	31.25/<br>31.25	7.81/<br>19.53	62.5/<br>78.13	13.68/<br>5.63	11.72/<br>11.72
		125	31.25	23.44	187.5	39.065	23.44	125	31.25	15.63	93.75	15.63	11.72

[a]4B, 5B, 6B mean *N*-benzyl-*N,N*-dimethyl-*N*-dodecylammonium bromide, *N*-benzyl-*N,N*-dimethyl-*N*-tetradecylammonium bromide, *N*-benzyl-*N,N*-dimethyl-*N*-hexadecylammonium bromide, respectively. Their preparation [2] and antimicrobial activities [3] has been described elsewhere.

Table S2. Minimum inhibitory concentrations (MIC) of the prepared compouds **4a – 6c** and selected standards **4 – 6B**[a] after 72h (yeasts)/168h (filamentous fungi) incubation. The activity *in vitro* was determined on a panel of four yeasts and three filamentous fungi.

**Microorganism**		**MIC [µmol/L]; 72h (yeasts)/168h (filamentous fungi) incubation**											
		**Compound**											
		**4a**	**5a**	**6a**	**4b**	**5b**	**6b**	**4c**	**5c**	**6c**	**4B**	**5B**	**6B**
Yeasts	*Candida parapsilosis sensu stricto* EXF-8411 (CAPA)	>1000	300	100	>1000	300	100	>1000	300	100	1000	300	1000
	Rhodotorula mucilaginosa EXF-8417 (RHMU)	300	30	100	500	100	100	500	100	100	500	100	500
	Exophiala dermatitidis EXF-8470 (EXDE)	400	0,03	65	1000	30	30	1000	100	30	500	100	200
	Aureobasidium melanogenum EXF-8432 (AUME)	300	0,03	30	300	30	30	100	30	30	200	100	65
Filamentous fungi	*Bisifusarium dimerum* EXF-8427 (BIDI)	>1000	>1000	500	>1000	>1000	500	>1000	>1000	500	>1000	1000	1000
	Penicillium chrysogenum EXF-1818 (PECH)	1000	300	300	1000	300	750	1000	100	300	650	650	650
	Aspergillus versicolor EXF-8692 (ASVE)	1000	300	100	>1000	100	200	1000	100	65	1000	650	1000

[a]4B, 5B, 6B mean *N*-benzyl-*N,N*-dimethyl-*N*-dodecylammonium bromide, *N*-benzyl-*N,N*-dimethyl-*N*-tetradecylammonium bromide, *N*-benzyl-*N,N*-dimethyl-*N*-hexadecylammonium bromide, respectively. Their preparation [2] and antimicrobial activities [3] has been described elsewhere

Table S3. Reduction factor of compounds **4a – 6c** and **4 – 6B** against Varicella zoster virus (VZV), ie. The difference of the values of 50% tissue culture infectious doses ($\Delta TCID_{50}$) of viral titre before and after 5 min exposition time.

Virus	$\Delta TCID_{50}$; 5 min exposure											
	Compound											
	4a	5a	6a	4b	5b	6b	4c	5c	6c	4B	5B	6B
VZV	0.83	1.34	0.69*	0.5	1.34	1*	0.5	0	1*	4.67	2.67	3.50*

Varicella zoster virus *(*5 min exposure to 0.01% compound; $\Delta TCID_{50}$)

*Varicella zoster virus *(*5 min exposure to 0.005% compound; $\Delta TCID_{50}$)

Table S4. Cytotoxicity of prepared QASs and their standards against mammalian cell line CHO-K1 (Chinese hamster ovary) cells and the Clog P^b.

Cell line	IC_{50} [µmol/L]; 24h incubation ± SEM											
	Compound											
	4a	5a	6a	4b	5b	6b	4c	5c	6c	4B	5B	6B
CHO-K1	23.845 ± 4.315	12.175 ± 0.295	9.884 ± 0.054	22.34 ± 1.31	12.475 ± 1.125	8.272 ± 1.602	28.29 ± 3.91	17.25 ± 1.9	13.065 ± 0.045	29 ± 1.2	24 ± 0.1	15 ± 1.4
Clog P^b	2.119	3.177	4.235	2.119	3.177	4.235	1.119	2.177	3.235	2.63	3.52	4.41

References

1. Gonzalez-Perez, A.; Varela, L.M.; Garcia, M.; Rodriguez, J.R. Sphere to rod transitions in homologous alkylpyridinium salts: a Stauff-Klevens-type equation for the second critical micelle concentration. *J Colloid Interface Sci* **2006**, *293*, 213-221, doi:10.1016/j.jcis.2005.06.026.
2. Kuca, K.; Marek, J.; Stodulka, P.; Musilek, K.; Hanusova, P.; Hrabinova, M.; Jun, D. Preparation of benzalkonium salts differing in the length of a side alkyl chain. *Molecules* **2007**, *12*, 2341-2347, doi:10.3390/12102341.
3. Benkova, M.; Soukup, O.; Prchal, L.; Sleha, R.; Eleršek, T.; Novak, M.; Sepčić, K.; Gunde-Cimerman, N.; Dolezal, R.; Bostik, V., et al. Synthesis, Antimicrobial Effect and Lipophilicity-Activity Dependence of Three Series of Dichained *N* -Alkylammonium Salts. *ChemistrySelect* **2019**, *4*, 12076-12084, doi:10.1002/slct.201902357.
